# Diversity of species and geographic distribution of tick-borne viruses in China

**DOI:** 10.3389/fmicb.2024.1309698

**Published:** 2024-02-27

**Authors:** Yi Wu, Qian Zhou, Meihan Mao, Huangliang Chen, Rui Qi

**Affiliations:** Institute of Microbiome Frontiers and One Health, School of Public Health, Lanzhou University, Lanzhou, Gansu, China

**Keywords:** tick-borne virus, tick, distribution, phylogenetic analysis, diversity

## Abstract

**Introduction:**

Tick-borne pathogens especially viruses are continuously appearing worldwide, which have caused severe public health threats. Understanding the species, distribution and epidemiological trends of tick-borne viruses (TBVs) is essential for disease surveillance and control.

**Methods:**

In this study, the data on TBVs and the distribution of ticks in China were collected from databases and literature. The geographic distribution of TBVs in China was mapped based on geographic locations of viruses where they were prevalent or they were detected in vector ticks. TBVs sequences were collected from The National Center for Biotechnology Information and used to structure the phylogenetic tree.

**Results:**

Eighteen TBVs from eight genera of five families were prevalent in China. Five genera of ticks played an important role in the transmission of TBVs in China. According to phylogenetic analysis, some new viral genotypes, such as the Dabieshan tick virus (DTV) strain detected in Liaoning Province and the JMTV strain detected in Heilongjiang Province existed in China.

**Discussion:**

TBVs were widely distributed but the specific ranges of viruses from different families still varied in China. Seven TBVs belonging to the genus *Orthonairovirus* of the family *Nairoviridae* such as Nairobi sheep disease virus (NSDV) clustered in the Xinjiang Uygur Autonomous Region (XUAR) and northeastern areas of China. All viruses of the family *Phenuiviridae* except Severe fever with thrombocytopenia syndrome virus (SFTSV) were novel viruses that appeared in the last few years, such as Guertu virus (GTV) and Tacheng tick virus 2 (TcTV-2). They were mainly distributed in the central plains of China. Jingmen tick virus (JMTV) was distributed in at least fourteen provinces and had been detected in more than ten species of tick such as *Rhipicephalus microplus* and *Haemaphysalis longicornis*, which had the widest distribution and the largest number of vector ticks among all TBVs. Parainfluenza virus 5 (PIV5) and Lymphatic choriomeningitis virus (LCMV) were two potential TBVs in Northeast China that could cause serious diseases in humans or animals. Ixodes persulcatus carried the highest number of TBVs, followed by *Dermacentor nuttalli* and *H. longicornis*. They could carry as many as ten TBVs. Three strains of Tick-borne encephalitis (TBEV) from Inner Mongolia Province clustered with ones from Russia, Japan and Heilongjiang Province, respectively. Several SFTSV strains from Zhejiang Province clustered with strains from Korea and Japan. Specific surveillance of dominant TBVs should be established in different areas in China.

## 1 Introduction

With the ever-changing global climate and ecology, the rapid spread of arthropod-borne diseases around the world has become a severe public health problem. Ticks are carriers of a wide range of pathogens including viruses, bacteria and parasites (Fang et al., 2015). The majority of ticks have to change 2 or 3 hosts throughout their whole life cycles, known as two-host ticks or three-host ticks, respectively. The pathogens are transmitted to hosts when ticks suck blood for energy and nutrients to grow (Simo et al., 2017; Boulanger et al., 2019). A variety of severe diseases to humans and animals are transmitted by ticks, such as Crimean Congo hemorrhagic fever, Tick-borne encephalitis, Severe fever with thrombocytopenia syndrome, Babesiosis and Anaplasmosis (Eisen et al., 2017; Tsao et al., 2021; El-Alfy et al., 2023). The transmission of tick-borne diseases is influenced by many factors such as animal reservoir ecology, environment and vector ecology, among which the environment plays an important role. Different species of ticks require different living environments, and their developmental stages, population distribution and abundance are also influenced by the climate (Gilbert, 2021; Couret et al., 2022). The species and health status of the animal hosts, the presence of symbionts, and tick-host interactions also modulate the tick microbiome. Due to the diversity of factors, tick-borne diseases are very difficult to prevent.

The viruses carried and transmitted by ticks are called tick-borne viruses which are very important among tick-borne pathogens. TBVs from at least two orders, nine families, and twelve genera are distributed in many countries around the world, such as Heartland virus, Bourbon virus and Powassan virus in the United States, Omsk hemorrhagic fever virus in Europe and Crimean Congo hemorrhagic fever virus (CCHFV)in Africa. Once transmitted to their susceptible hosts, these viruses rapidly begin to replicate and invade target organs or cells, resulting in serious diseases in humans or animals (Owen et al., 2021). With the development of pathogen detection and high-throughput sequencing technologies, more and more novel TBVs or novel genotypes of TBVs have been discovered through either isolating in patients after tick bites or assembling and annotating by metagenomic next-generation sequencing (Damian et al., 2020; Madison-Antenucci et al., 2020; Piantadosi and Kanjilal, 2020).

The emergence of novel TBVs deserves more attention (Park et al., 2020). In recent years, there have been numerous incidents of people being bitten by ticks and being infected with pathogens that have led to serious illnesses and even deaths (Dragonjic et al., 2022; Jumpertz et al., 2023). China covers various lands with large environmental differences between regions. The widespread distribution of ticks makes China a “natural greenhouse” for the spread and evolution of TBVs. However, most of the previous reviews on tick-borne pathogens in China focused on bacteria, parasites, and viruses together, with viruses only having a small portion (Wu et al., 2013; Luan et al., 2023). Although some studies only focus on TBVs in China, they were not comprehensive due to geographical limitations (Bai et al., 2023; Cai et al., 2023). The overall distribution of TBVs in China is not clear. Due to the rapid evolution, mutation, and recombination of viruses, the database of TBVs needed to be updated continuously even though TBVs were considered to be slower evolving among the arboviruses. Therefore, this study aimed to analyze the prevalence of TBVs in China, including the species of virus vectors and their geographic distribution.

## 2 Materials and methods

### 2.1 Data collection

Firstly, data on TBVs in China was from a database named “global dataset of sequence, diversity and biosafety recommendation of arbovirus and arthropod-specific virus” recently published by Huang et al. This database contains taxonomic, genomic, sequence, host, biosafety, and isolation source information for arboviruses and arthropod-specific viruses (Huang Y et al., 2023). The literature on TBVs in China was searched in PubMed, Web of Science, and CNKI with the keywords “China” and “tick-borne viruses,” “TBVs” or specific virus names such as “Crimean Congo hemorrhagic fever virus” or “CCHFV” to collect more data. All records of viruses in this study were detected by PCR, nested PCR, RT-PCR or isolated and cultured. Those that only were identified in high-throughput sequencing but not detected in human or animal hosts were excluded. The distribution of ticks in this study was from a dataset of distribution and diversity of ticks in China and published literature on tick species (Chen et al., 2010; Zhang G et al., 2019; Zhang Y et al., 2019; Yang et al., 2020; Zhao et al., 2021).

### 2.2 Mapping the distribution of TBVs

The geographic distribution of TBVs in China was mapped based on geographic locations of viruses where they were prevalent or they were detected in vector ticks. In addition to the general distribution map of TBVs, the distribution of different virus families was mapped separately. A land-cover map of China was downloaded through the National Earth System Science Data Center, National Science & Technology Infrastructure of China^1^ as a base map for the geographic distribution of the TBVs (Wang et al., 2019). There are ten major categories of land cover information including Cultivated land, Forest, Bush, Grassland, Water, Wetland, Glacier, Artificial land, Bare soil and Tundra. The distribution of all ticks that are carriers of a particular virus was mapped and compared with the geographic distribution of the virus. All maps were drawn using ArcGIS 10.7 software.

### 2.3 Phylogenetic analysis of TBVs

TBVs sequences were collected from The National Center for Biotechnology Information (NCBI). The S-segment of CCHFV, NSDV, SFTSV, as well as DTV, the first segment of JMTV, and the whole genomes TBEV were aligned using MUSCLE version 3.8.31 software. Phylogenetic trees of NJ were constructed using the MEGA 11 software with the Kimura two-parameter model and 1,000 Bootstrap replications.

## 3 Results

Eighteen viruses from eight genera of five families were identified in China (Table 1). Some of these viruses were also widely distributed in other areas of the world, some were only found in China. More than sixteen species of ticks from six genera were found to carry or transmit TBVs in China, most of which were from the family *Ixodidae.* TBVs found and reported only by high-throughput sequencing were listed in Supplementary Table S1.

**Table 1:** Tick-borne viruses in China.

Family	Genus	Species	Host	Vector	Geographical distribution in China	Geographical distribution in the world	References
*Nairoviridae*	*Orthonairovirus*	Nairobi sheep disease virus	Human, sheep, cattle	*H. longicornis*	Hubei, Jilin, Liaoning	China, India, Kenya, Somalia, Uganda, Tanzania, Sri Lank	Bai et al. (2023), Gong S et al. (2015), Gong Z et al. (2015), Marczinke and Nichol (2002), and Yang et al. (2019)
		Tacheng tick virus 1	Human, cattle, sheep	*D. marginatus, D. nuttalli, D. silvarum, Hy. asiaticum*	XUAR	China, Turkey, Kazakhstan	Dincer et al. (2023), Ji et al. (2023), Liu et al. (2020), and Zhang et al. (2021)
		Tamdy virus	Human	*Hy. asiaticum*	XUAR	China, Kazakhstan, Kyrgyzstan, Turkmenistan, Uzbekistan	Lvov et al. (1976b), Moming et al. (2021), and Zhou et al. (2019)
		Crimean-Congo hemorrhagic fever virus	Human, sheep, rodent, hares, etc.	*Hy. asiaticum, Hy. marginatum, Hy. dromedraii, D. nuttalli*	XUAR, Inner Mongolia, Yunnan, etc.	China, Africa, the Middle East Asia, Southern and Eastern Europe	Fereidouni et al. (2023), Guo et al. (2017), Kong et al. (2022), Li et al. (2020), Moming et al. (2018), Sun et al. (2009), Xia et al. (2011), Zhang Y et al. (2018), and Zhou et al. (2013b)
		Songling virus	Human, rodent	*I. crenulatus, I. persulcatus, H. longicornis*	Jilin, XUAR, Heilongjiang, Inner Mongolia	China	Cai et al. (2023), Ji et al. (2023), and Ma et al. (2021)
		Beiji nairovirus	Human, sheep, cattle	*I. persulcatus, I. crenulatus, D. silvarum, H. concinna*, etc.	Inner Mongolia, Heilongjiang, Jilin	China	Liu et al. (2022) and Wang YC et al. (2021)
		Yezo virus	Human	*I. persulcatus*	Inner Mongolia, Heilongjiang, Jilin	China, Japan	Kodama et al. (2021) and Lv et al. (2023)
*Phenuiviridae*	*Bandavirus*	Severe fever with thrombocytopenia syndrome virus	Human, cattle, sheep, rodent, hedgehogs, mephitine, etc.	*H. longicornis, H. conicinna, H. japonica, I. persulcatus, R. microplus*, etc.	Hubei, Henan, Shandong, Zhejiang, Shaanxi, Anhui, Guizhou, XUAR, etc.	China, South Korea, Japan, Vietnam, Pakistan	Fujikawa et al. (2021), Han et al. (2018), Kim et al. (2018), Li J et al. (2022), Sun RX et al. (2017), Sun Y et al. (2017), Tao et al. (2021) Tian et al. (2017), Wen et al. (2014), Yoshikawa et al. (2015), Zhang et al. (2014), Zhao et al. (2012), Zhuang et al. (2018), and Zohaib et al. (2020)
		Guertu virus	Human, rodent	*D. nuttalli*	XUAR	China	Shen et al. (2018)
	*Phlebovirus*	Mukawa virus	Yezo deer, raccoons	*I. persulcatus, H. concinna*	Heilongjiang	China, Japan	Qin et al. (2023), Torii et al. (2019), and Wang et al. (2022)
	*Uukuvirus*	Dabieshan tick virus	Sheep	*R. microplus, H. longicornis, R. haemaphysaloides*	Yunnan, Guizhou, Zhejiang, Shandong	China, Japan	Ma et al. (2022), Shao JW et al. (2021), Shao L et al. (2021), Shao et al. (2020), Zhu et al. (2020), Wang A et al. (2021), and Mekata et al. (2023)
		Tacheng tick virus 2	Human	*D. nuttalli, D. marginatus, D. silvarum, Hy. asiaticum*	XUAR	China, Turkey, Kazakhstan	Dincer et al. (2022), Dong et al. (2021), and Jia et al. (2022)
*Flaviviridae*	*Orthoflavivirus*	Tick-borne encephalitis virus	Human, canine, marmot	*I. persulcatus, D. silvarum, D. nuttalli*	Inner Mongolia, Heilongjiang, Jilin, etc.	China, Russia, Germany, Japan, Netherlands, The United Kingdom, etc.	Chen et al. (2019), Holding et al. (2020), Jahfari et al. (2017); Li J et al. (2022), Li XH et al. (2022), and Takahashi et al. (2020)
		Karshi virus	Human, sheep, marmot	*Hy. asiaticum, Hy. dromedraii, D. nuttalli, O. papillipes*	XUAR	China, Uzbekistan	Bai et al. (2022) and Lvov et al. (1976a)
	*-*	Jingmen tick virus	Human, cattle, bat, rodent	*R. microplus, R. sanguineus, H. longicornis, H. campanulata, H. flava, H hystricis, I. granulatus, I. persulcatus, I sinensis, D. nuttalli, A javanense, A. testudinarium*	Hubei, Heilongjiang, Zhejiang, Yunnan, Guizhou, Henan, Shandong, Anhui, Fujian, etc.	China, Japan, Turkey, Kenya, etc.	Dincer et al. (2023), Huang L et al. (2023), Huang Y et al. (2023), Kobayashi et al. (2021), Ogola et al. (2022), Wu et al. (2023); Yu et al. (2020), and Zhang et al. (2022)
		Alongshan virus	Human, sheep, cattle	*I. persulcatus, H. conicinna*	Inner Mongolia, Heilongjiang	China, Russia, Finland, Switzerland	Kholodilov et al. (2020), Kuivanen et al. (2019), Stegmueller et al. (2023), and Wang et al. (2019a,b)
	*Hepacivirus*	Bovine hepacivirus	Cattle	*R. microplus*	Heilongjiang, Guangdong	China, Italy, the United States, etc.	Guo et al. (2022) and Yuan et al. (2023)
*Paramyxoviridae*	*Orthorubulavirus*	Parainfluenza virus 5	Human, canine, rodent	*I. persulcatus, D. silvarum*	Heilongjiang	China	Yang et al. (2022)
*Arenaviridae*	*Mammarenavirus*	Lymphocytic choriomeningitis virus	Human, rodent	*D. nuttalli, D. silvarum, H. longicornis, I. persulcatus*	Inner Mongolia, Heilongjiang, Jilin	China	Zhang L et al. (2018) and Zhang Y et al. (2018)

### 3.1 Nairoviridae

Seven TBVs are belonging to the genus *Orthonairovirus* of the family *Nairoviridae* in China. CCHFV, one of the most notable tick-borne viruses of the *Nairoviridae*, is widely distributed in the world and has been reported in Asia, Africa and Europe (Serretiello et al., 2020). The earliest record of CCHFV reported in China could be traced back to an outbreak of hemorrhagic fever in the southern XUAR in 1968, which was the reason that CCHFV was named Xinjiang hemorrhagic fever in China (Saijo, 2007). A study reported that more than 200 cases of CCHFV had occurred in China with more than 50 deaths as of 2003 (Fereidouni et al., 2023). A population serologic study for CCHFV reported that seropositive cases could be found in northeastern, northwestern, and southern China, but no cases of human infection had been detected outside of XUAR (Teng et al., 2022). *Hyalomma* spp. particularly *Hyalomma asiaticum* are the main vectors for the transmission of CCHFV. NSDV could cause zoonotic disease and was first identified in 1910 in sheep and goats in Kenya. In the past, East Africa was the main endemic area, but it was gradually being reported in Asia, including India and Sri Lanka (Krasteva et al., 2020). The first detection of NSDV in ticks in China was in *Haemaphysalis longicornis* collected from cattle and sheep in Jilin and Liaoning Provinces in 2015. Its genotype was different from other strains in Africa and Asia (Gong S et al., 2015). NSDV was also found in Hubei Province (Yang et al., 2019). Subsequently, NSDV was found by high-throughput sequencing in Liaoning Province with the same strain as previously reported in Hubei Province (Bai et al., 2023). The current records of NSDV in China were all found in *H. longicornis*.

According to the taxonomy of the order *Bunyavirales* updated by the International Committee on Taxonomy of Viruses (ICTV) in 2019, Tamdy virus (TAMV) and Tacheng tick virus 1 (TcTV-1) were both members of the species *Tamdy orthonairovirus* (Abudurexiti et al., 2019). TAMV has appeared frequently in Asia since it was first detected in Uzbekistan in 1971. It was not reported in China until it was detected in 2019 in hard ticks collected from Bactrian Camels in XUAR (Lvov et al., 1976b; Zhou et al., 2019). Evidence of human infection of TAMV was provided by the presence of TAMV IgG in humans from XUAR (Moming et al., 2021). TcTV-1 was first detected by high-throughput sequencing of *Dermacentor marginatus* in XUAR in 2015 (Li et al., 2015). Researchers isolated TcTV-1 in the cerebrospinal fluid, throat swabs, and urine of a woman engaged in agricultural activities in the XUAR who developed fever and rash symptoms after being bitten by a tick (Liu et al., 2020). The seroprevalence of TcTV-1 in the local population was as high as 15% and the serum samples from cattle and sheep were also partially positive by RT-PCR. TCTV-1 was found in 4 native tick species, of which *D. marginatus* had the highest positive rate. A study reported a man who developed clinical symptoms after a tick bite in XUAR and TcTV-1 and *Rickettsia raoultii* were detected in his blood samples by RT-PCR, he was finally diagnosed with severe acute meningitis co-infected with *Rickettsia raoultii* and TcTV-1 (Zhang et al., 2021). TcTV-1 infection in animals has been detected in giant gerbils from XUAR, and Songling virus (SGLV) was also detected (Ji et al., 2023). SGLV was a new *Orthonairovirus* first identified in 2021 in a male farmer bitten by a tick in Heilongjiang Province, with genomic and morphological characteristics similar to other *Orthonairovirus*. It was phylogenetically related to members of *Tamdy orthonairovirus* and was positive in 6.38% of local patients hospitalized for tick bites. SGLV was also found in local *Ixodes crenulatus, Ixodes persulcatus, Haemaphysalis concinna,* and *H. longicornis*. Subsequently, some researchers annotated the sequence of SGLV in the metagenomic sequencing of ticks from northeastern China (Liu et al., 2022; Cai et al., 2023; Ji et al., 2023). Beiji nairovirus (BJNV), also a new nairo-like virus, was first mentioned in high-throughput sequencing, and then it was detected in a febrile patient in Inner Mongolia Province. Researchers identified BJNV infections in several tick-bitten patients in Heilongjiang and Jilin Provinces, and BJNV was also detected in local ticks, including *I. persulcatus, I. crenulatus, Demacentor silvarum, D. nuttalli, H. concinna* and *H. longicornis* (Wang YC et al., 2021; Liu et al., 2022). Yezo virus (YEZV) is a new *Orthonairovirus* first detected in Japan in 2019 and is currently only reported in Japan and China (Kodama et al., 2021). The first detection in China was in a man bitten by an *I. persulcatus* tick from Inner Mongolia Province. YEZV was also detected in *I. persulcatus* collected in Heilongjiang, Inner Mongolia, and Jilin Provinces. YEZV was genetically related to Sulina virus found in *Ixodes ricinus* from Romania in phylogenetic analysis, but YEZV was in a unique cluster of the phylogenetic tree with TAMV, SGLV, TcTV-1 (Lv et al., 2023). Notably, a novel virus strain was detected from *D. silvarum* in Jilin Province recently. Phylogenetic analyses showed that it was in the same branch as SGLV, but homology analyses with the SGLV strain showed that this strain might be a novel *Orthonairovirus*, and researchers have named it Antu virus (Li et al., 2023).

### 3.2 Phenuiviridae

The family *Phenuiviridae* in this study included four TBVs, of which the most widely distributed in China was SFTSV of the genus *Bandavirus*. SFTSV is a new virus of the order *Bunyavirus* discovered in China in 2011. The first strain was isolated from a man in Henan Province, and researchers subsequently detected SFTSV in several febrile patients, as well as in *H. longicornis* collected from domestic animals in the area where the patients lived. SFTSV was also reported in South Korea, Japan, Vietnam and Pakistan in recent years (Yu et al., 2011; Han et al., 2018; Kim et al., 2018; Zohaib et al., 2020; Fujikawa et al., 2021; Tran et al., 2022). *H. longicornis* was the most common vector of SFTSV, and it was found that SFTSV could be transmitted vertically in *H. longicornis*. SFTSV could be detected in eggs laid by SFTSV-carrying females as well as in mature larvae, nymphs, and adults after transstadial transmission (Zhuang et al., 2018). From literature published since 2011, it could be found that SFTSV had a very wide geographical distribution in China and its vectors also included *Ixodes* spp. and *Rhipicephalus* spp. (Pan et al., 2013; Sun Y et al., 2017). In contrast to other TBVs, SFTSV could not only be transmitted by ticks but also be transmitted directly from person to person through direct contact with the infected blood (Chen et al., 2013; Gong et al., 2018; Fang et al., 2021). Aerosol transmission has also been suggested as a potential human-to-human transmission route of SFTSV (Gong Z et al., 2015). As another member of the genus *Bandavirus*, GTV is a new tick-borne *Bandavirus* detected in 2018 from *D. nuttalli* and human serum samples in XUAR. It is genetically related to SFTSV and Heartland virus by phylogenetic analyses and may have a similar RNA replication mechanism to SFTSV (Shen et al., 2018). Mukawa virus (MKWV) was first detected in *I. persulcatus* in Japan in 2018, and researchers found that *I. persulcatus* and *H. concinna* in Heilongjiang Province carried MKWV and were capable of infecting mammals, but it had not yet been reported in other countries or regions (Wang et al., 2022; Qin et al., 2023). It was curious that MKWV was not genetically similar to any other tick-borne *Phenuiviridae* viruses on the phylogenetic tree, instead, it was related to viruses transmitted by mosquitoes and sandflies (Matsuno et al., 2018). DTV was initially identified in high-throughput sequencing of *H. longicornis* in Hubei Province. It exists in several provinces in southern China. DTV genetically clustered into a branch of the Yongjia tick virus (Li et al., 2015; Shao et al., 2020; Zhu et al., 2020; Wang A et al., 2021; Ma et al., 2022). However, there was no evidence of DTV infection in humans. All DTV strains in China were found in ticks, except a report of detection in sheep serum in Shandong Province (Shao L et al., 2021). TcTV-2 was detected by high-throughput sequencing and classified into the family of *Phenuiviridae*, without being assigned to a specific genus. There was some evidence that humans and several ticks could be infected with TcTV-2. It also appeared in regions outside of China including Turkey and Kazakhstan (Li et al., 2015; Dong et al., 2021; Dincer et al., 2022; Jia et al., 2022).

### 3.3 Flaviviridae

The family *Flaviviridae* includes many species of arboviruses, such as Dengue virus, a mosquito-borne virus that poses a serious threat to humans. Those tick-borne flaviviruses of the family *Flaviviridae* made a group called TBEV serocomplex group, which includes TBEV, Omsk haemorrhagic fever virus (OHFV), Powassan virus (POWV) and Karshi virus (KSIV) (Grard et al., 2007). TBEV was one of the most widespread viruses of the TBEV serocomplex group in the world, especially in Europe. People in Japan, Korea and China were also at risk of TBEV infection (Lindquist and Vapalahti, 2008). There were three subtypes of TBEV including the Far Eastern (TBEV-FE) which was the most prevalent in China, the Siberian (TBEV-Sib) and the European (TBEV-EU), but a new subtype named the Himalayan TBEV was found in China (Phipps and Johnson, 2022). At present, the forested areas of northeastern China have a high prevalence of TBEV, so previous studies on TBEV in China mainly focused on this region (Chen et al., 2019; Li XH et al., 2022). Karshi virus (KSIV) was first identified in *Ornithodoros papillipes* in Uzbekistan. KSIV was detected in *Hy. dromedraii, Hy. asiaticum,* and *D. nuttalli* from 14 sampling sites in XURA with a minimum infection rate of 4.96%. Researchers successfully isolated virus particles from *Hy. asiaticum* and then found KSIV in local domesticated sheep and wild-caught greater gerbils (Lvov et al., 1976a; Bai et al., 2022). They also demonstrated the existence of serological cross-reactivity between TBEV and KSIV, with the antibodies against these two viruses that could recognize each other’s antigens. JMTV was a novel segmented tick-borne *flavivirus* that had been reported in several provinces in China, it was first identified by high-throughput sequencing of ticks in Hubei Province in 2014 (Qin et al., 2014; Guo et al., 2020; Yu et al., 2020; Pang et al., 2022; Zhang et al., 2022; Qu et al., 2023; Wu et al., 2023). JMTV was also found in several countries around the world. In addition to ticks, mosquitoes were also reported to be infected with JMTV (Ladner et al., 2016). There already had proof of JMTV infection in humans in China, as a study detected JMTV in a skin biopsy sample taken from a patient’s tick bite site (Jia et al., 2019). Recently, a new type of JMTV was discovered by high-throughput sequencing in ticks in Sichuan Province and named Sichuan tick virus (Huang L et al., 2023). Alongshan virus (ALSV) was also a novel segmented tick-borne virus isolated from a patient presenting with unexplained fever in Inner Mongolia Province in 2019 and was classified in the Jingmenvirus group because of its genetic similarity to JMTV (Wang et al., 2019a). Other local febrile patients with a history of tick bites were also confirmed to be infected with ALSV. Besides, the prevalence of ALSV positivity in ticks collected at the workplace of these patients or at the location where they were bitten was 6.5% (Wang et al., 2019b). ALSV has been reported in Europe (Kuivanen et al., 2019; Kholodilov et al., 2020; Stegmueller et al., 2023). Bovine hepacivirus (BovHepV) was a genus of hepatitis viruses found in *R. microplus* in Guangdong and Heilongjiang Provinces, which mainly infected cattle with severe impacts on the farming industry. But no human infections were documented (Shao JW et al., 2021; Guo et al., 2022; Yuan et al., 2023).

### 3.4 Potential tick-borne viruses of other virus families

PIV5 of the family *Paramyxoviridae* could cause disease in humans and animals (Wang et al., 2023). The detection of PIV5 in arthropods had not been documented until it was found in ticks from Jilin Province (Yang et al., 2022). LCMV of the family *Arenaviridae* was a group of rodent-borne viruses that could cause neurological disorders in humans (Barton et al., 2002). LCMV was isolated from ticks in northeastern China which was the first report of LCMV in China. It has been predicted that LCMV in ticks and transmitted among ticks in Jilin Province might have occurred in the 1970s or 1980s (Zhang L et al., 2018). Both PIV5 and LCMV were considered zoonotic viruses, they only were detected or isolated from ticks in China. There were no documented cases of ticks as a vector to infect humans, so they should be treated as potential tick-borne viruses.

### 3.5 Geographical distribution of tick-borne viruses in China

**Figure 1:**
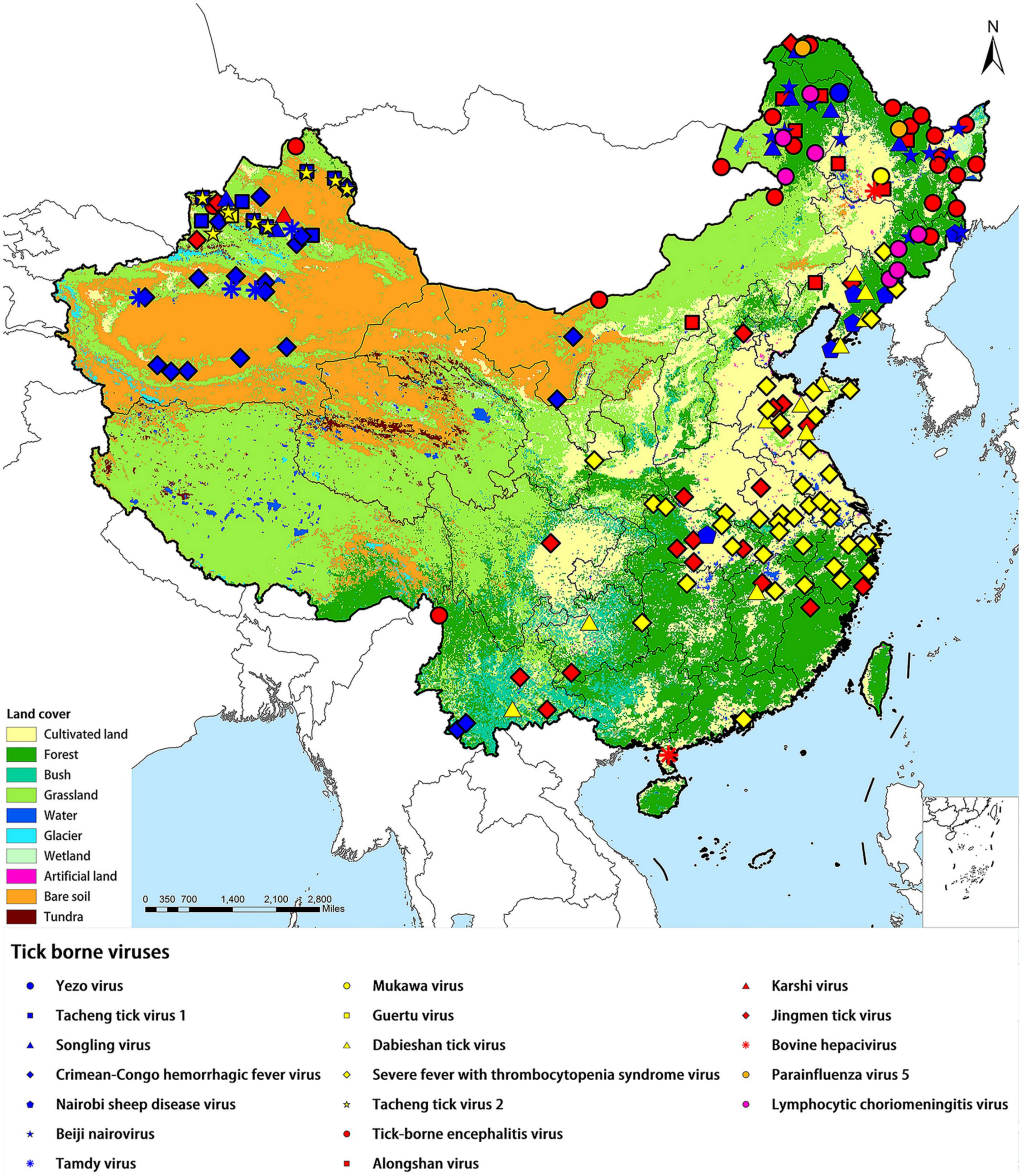
Distribution of tick-borne viruses in China. China’s land cover map was used as a background map. Blue squares represented the *Nairoviridae* family, yellow squares represented the *Phenuiviridae* family, and red represented the *Flaviviridae* family.

**Figure 2:**
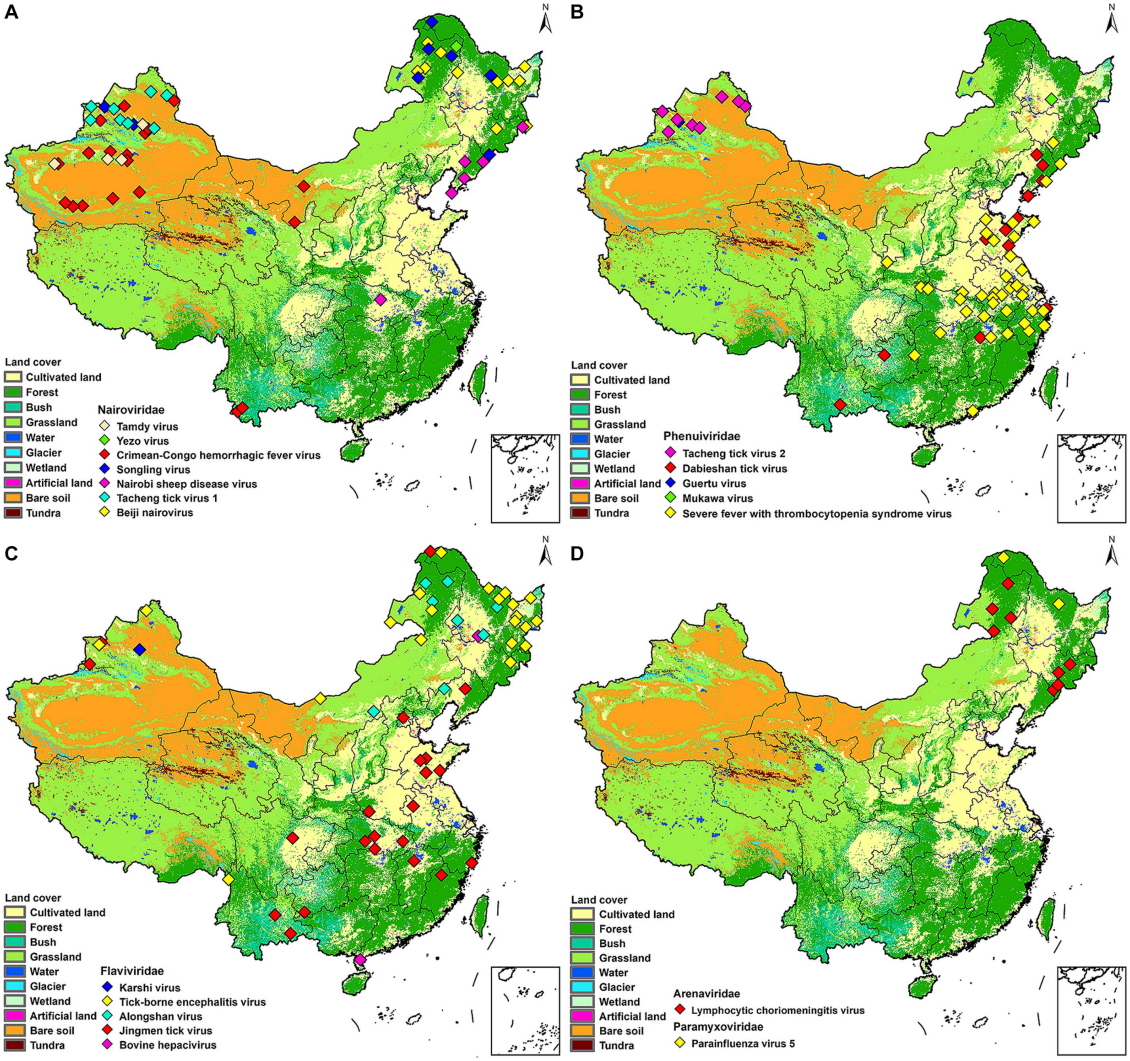
Mapping of tick-borne viruses by family. China’s land cover map was used as a background map. **(A)** *Nairoviridae*, **(B)** *Phenuiviridae*, **(C)** *Flaviviridae*, **(D)** *Paramyxoviridae* and *Arenaviridae*. Different colors were used in each figure to denote different viruses.

All the TBVs mentioned in this paper were widely distributed in China including northeastern forested areas, coastal areas, and northwestern interior. XUAR currently has the largest number of TBVs, containing eight viruses from the family *Nairoviridae* (CCHFV, TAMV, TcTV-1, and SLTV), the family *Flaviviridae* (TBEV, KSTV, and JMTV), and the family *Phenuiviridae* (GTV). The next richest group of TBVs was found in northeastern Inner Mongolia and Heilongjiang Provinces, where all five virus families were present, with the family *Flaviviridae* and the family *Nairoviridae* predominating. Another area of viruses affected was central China, where most of the viruses were from the family *Phenuiviride*. TBVs in China were less studied focusing on the Tibetan Plateau, northwestern China outside of XUAR, such as Gansu Province and western Inner Mongolia Province, and southern China (Figure 1). TBVs of the family *Nairoviridae* were distributed in most parts of XUAR and northeastern China (Figure 2A). Many provinces in China had human serologic evidence suggesting the presence of CCHFV, but the provinces where CCHFV was detected in ticks was only XUAR, Yunnan and Inner Mongolia Provinces (Teng et al., 2022). CCHFV was mainly distributed in the grass-covered areas around the Tarim Basin and the Tianshan Mountains in XUAR. In addition, it was reported to be detected in ticks and camels from the Alashan League of Inner Mongolia Province (Xia et al., 2011; Kong et al., 2022). Four species of ticks could carry or transmit CCHFV including *Hy. dromedraii, Hy. asiaticum, Hy. marginatum* and *D. nuttalli* which were mainly found in northwestern China (Supplementary Figure S1A). TCTV-1 was only in the Tacheng and Altay regions of XUAR, and geographically clustered in the Tianshan Mountains. TCTV-1 could be transmitted by *D. marginatus, D. nuttalli, D. silvarum,* and *Hy. asiaticum*. Ticks of the *Dermacentor* spp. were the main carriers of TCTV-1 with a higher rate. These ticks were widely distributed in northern China, but there were no records of TCTV-1 except in XUAR (Supplementary Figure S1B). TAMV appeared sporadically in the Kashi, Aksu, and Tacheng regions, with three reported sites around the Tarim Basin and one at the junction of the TianShan Mountains and the Junggar Basin (Supplementary Figure S1C). SLGV was also reported from the Tianshan Mountains in XUAR, although most SLGV in China were now reported from northeastern China, clustering in the forest of the Greater Khingan Mountains. Ticks of *Ixodes* spp. were the main vectors of SLGV, and all records of SGLV in China were in the *I. persulcatus* distribution area, with partial overlap of the *I. crenulatus* or the *H. longicornis.* Distribution area (Supplementary Figure S1D). NSDV was mainly clustered in Liaoning and Jilin Provinces, except for one sampling site reported in Suizhou City in Hubei Province (Supplementary Figure S1E). BJNV was distributed in northeastern Inner Mongolia Province and eastern Heilongjiang Province (Supplementary Figure S1F).

Both GTV and MKWV were only recorded once in Usu City and Heilongjiang Province, respectively (Figure 2B). SFTSV were reported in 12 provinces, with Xinyang City in Henan Province having the highest number of cases, and Hubei, Anhui, Zhejiang, and Shandong Provinces all having high prevalence of SFTSV (Miao et al., 2020). The vectors of SFTSV included five species of ticks, *H. longicornis* was its confirmed vector, but SFTSV were also detected in *H. conicinna, H. japonica, I. persulcatus, and R. microplus*. However, most of the SFTSVs were still clustered in the *H. longicornis* distribution areas (Figure 2B; Supplementary Figure S2A). DTV is now distributed in six provinces of China, with a high prevalence in Shandong and Liaoning Provinces, partially overlapping with SFTSV. It was also found in forested and shrub-covered areas of Yunnan and Guizhou Provinces. Two ticks of *Rhipicephalus* spp. and *H. longicornis* were the vectors for DTV (Figure 2B; Supplementary Figure S2B). TcTV-2 is currently distributed in XUAR, clustered around the Tianshan Mountains, and has the same vectors and distribution characteristics as TcTV-1, although they are not in the same family (Figure 2B; Supplementary Figure S2C).

JMTV was the most widely distributed virus in our study. It was dispersed in 14 provinces of China, from Mohe City in Heilongjiang Province in the north to Chabuchar Xibe and Bole counties in XUAR in the west, to the coastal areas of Fujian in the east, and Wenshan County in Yunnan Province in the south. However, the areas were mainly in central China, with Hubei and Shandong Provinces reporting more than others (Figure 2C; Supplementary Figure S3A). In addition to the wide geographic distribution, JMTV had the widest variety of vectors. As the dominant tick species varied among regions, the ticks that carried JMTV varied from different regions, including at least 5 genera and 11 species, which were distributed throughout China. TBEV was clustered in Heilongjiang and northeastern Inner Mongolia Provinces, mostly in forest-covered areas. The vector of most TBEV was *I. persulcatus*, but *D. nuttalli* and *D. silvarum* could also carry it in some studies. In addition to the large number of *I. persulcatus* in the forested areas of Northeast China, it was also found in the Karshi region of XUAR, but there were fewer cases of TBEV in XUAR (Figure 2C; Supplementary Figure S3B). ALSV was also a young virus detected recently, with *I. persulcatus* and *H. conicinna* were its vectors, and the only areas where both ticks were distributed were northern XUAR and southern Heilongjiang Province (Figure 2C; Supplementary Figure S3C).

PIV5 was detected only in Mohe City and Yichun City in Heilongjiang Province (Figure 2D; Supplementary Figure S4A). LCMV could be carried by 4 species of ticks, which were densely distributed in the XUAR and Shaanxi Provinces. LCMVs were detected only in the forested areas of the Greater Khingan Mountains and the Changbai Mountains (Figure 2D; Supplementary Figure S4B).

### 3.6 Phylogenetic analysis of tick-borne viruses

**Figure 3:**
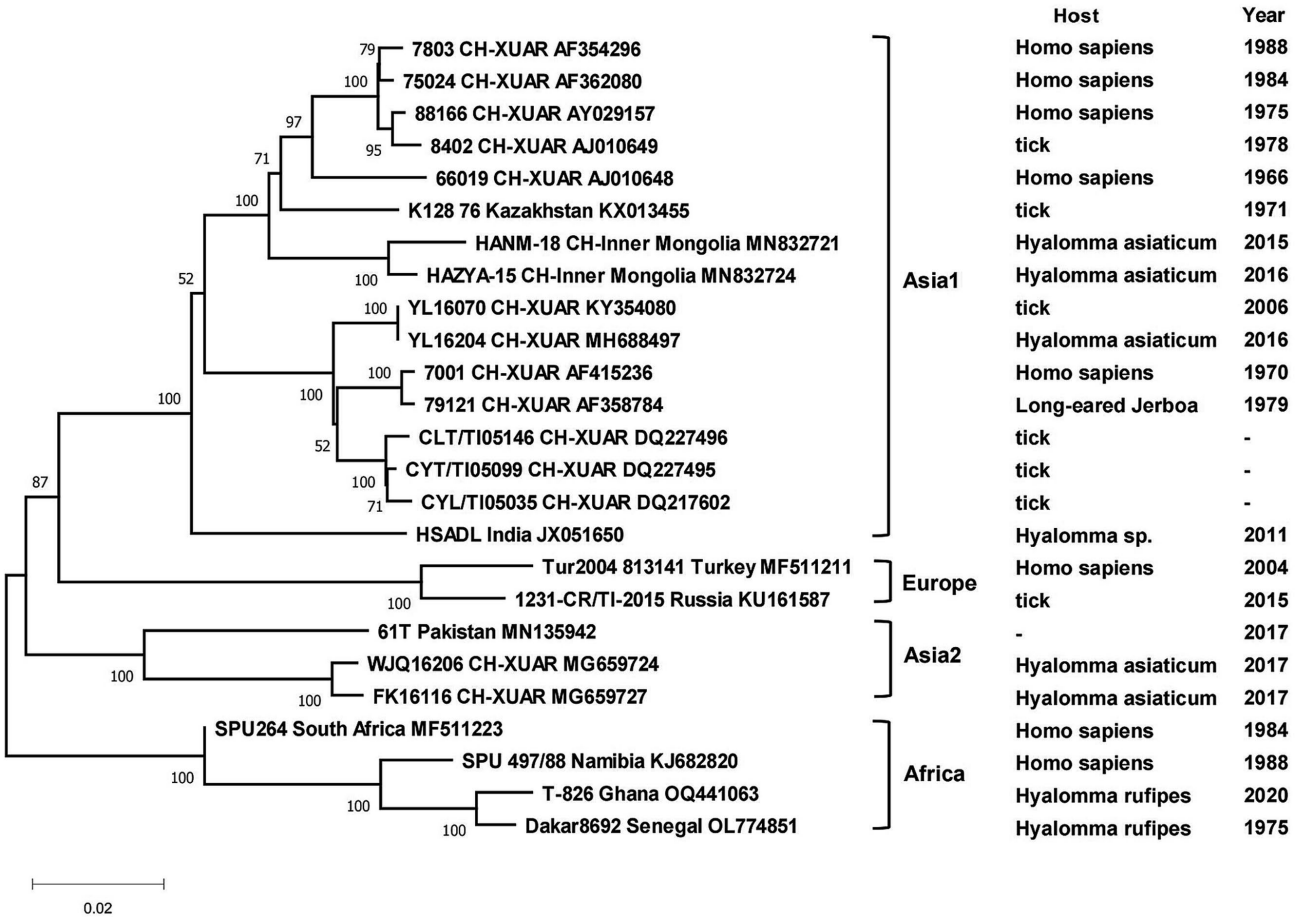
Phylogenetic tree of the S segment of Crimean Congo hemorrhagic fever virus. Each sequence was labeled with its host and sampling time (same for the later figures). The missing data in the figure were not recorded in NCBI.

**Figure 4:**
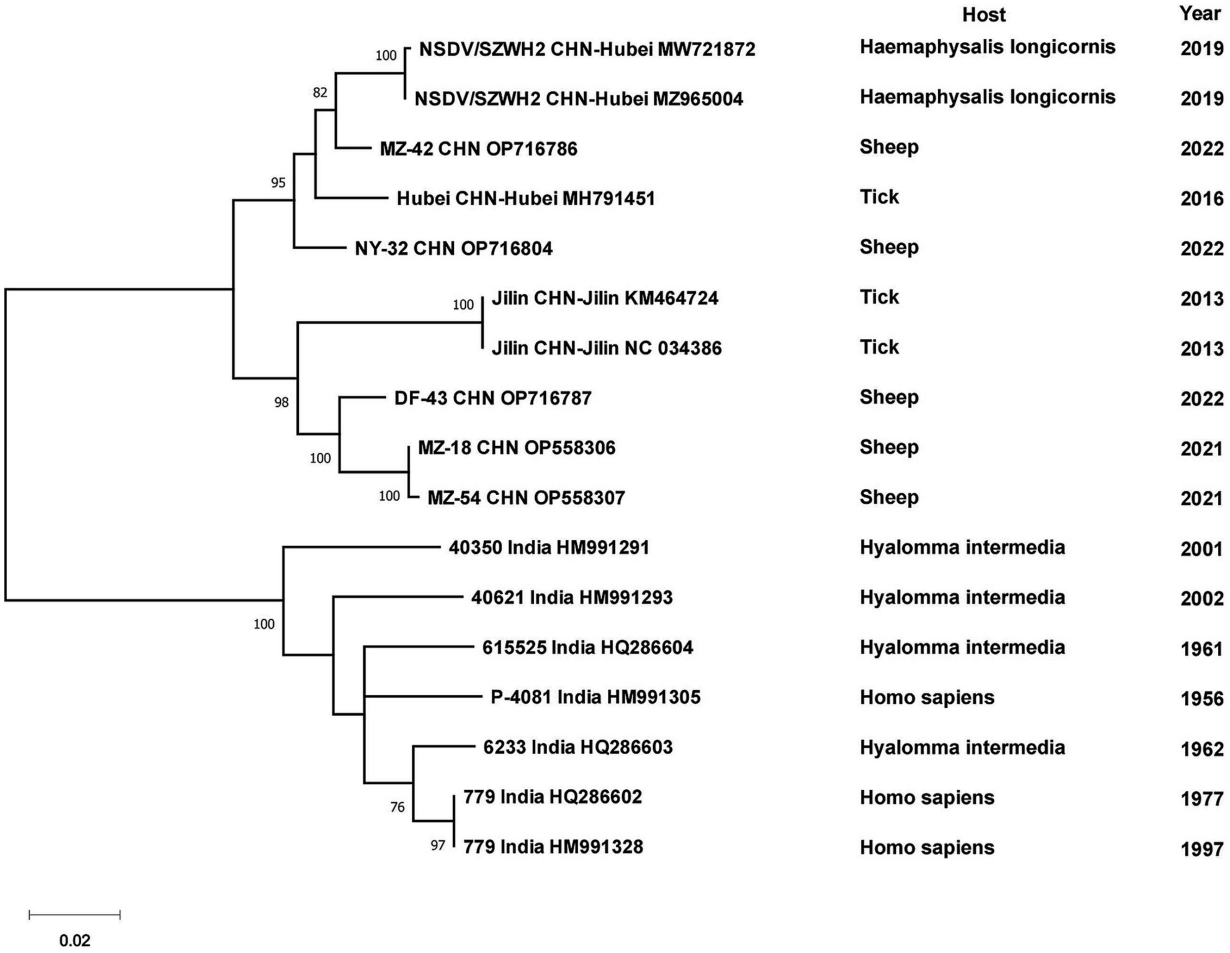
Phylogenetic tree of the S segment of Nairobi sheep disease virus.

Phylogenetic analyses of the sequences collected in NCBI from different areas of China were performed to analyze the genetic relationships among the viruses. CCHFV was discovered early and classified into three genotypes: Asian, European and African (Serretiello et al., 2020). CCHFV in China was divided into two Asian aggregation branches. Strains from XUAR clustered together with two strains detected from *Hy. asiaticum* in Inner Mongolia Province. Although the strains found in XUAR had various host species, they remained genetically clustered. The other branch was a cluster of two strains detected in *Hy. asiaticum* in XUAR and strains from Pakistan (Figure 3). There were not many strains of NSDV in China, but they formed two discrete branches from the Indian strains (Figure 4).

**Figure 5:**
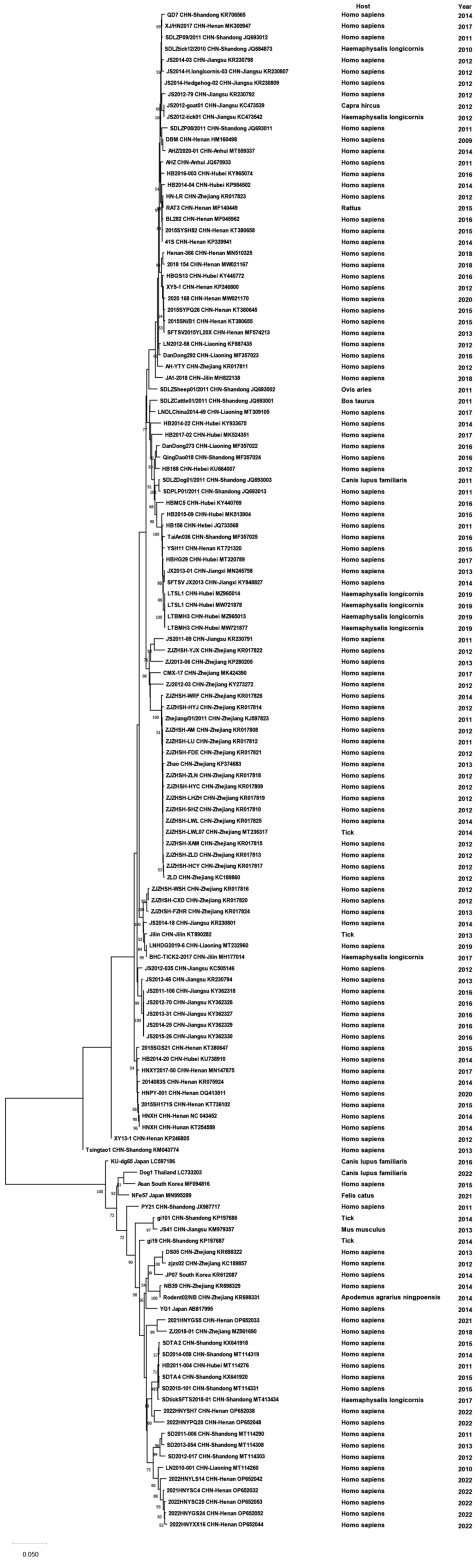
Phylogenetic tree of the S segment of Severe fever with thrombocytopenia syndrome virus.

**Figure 6:**
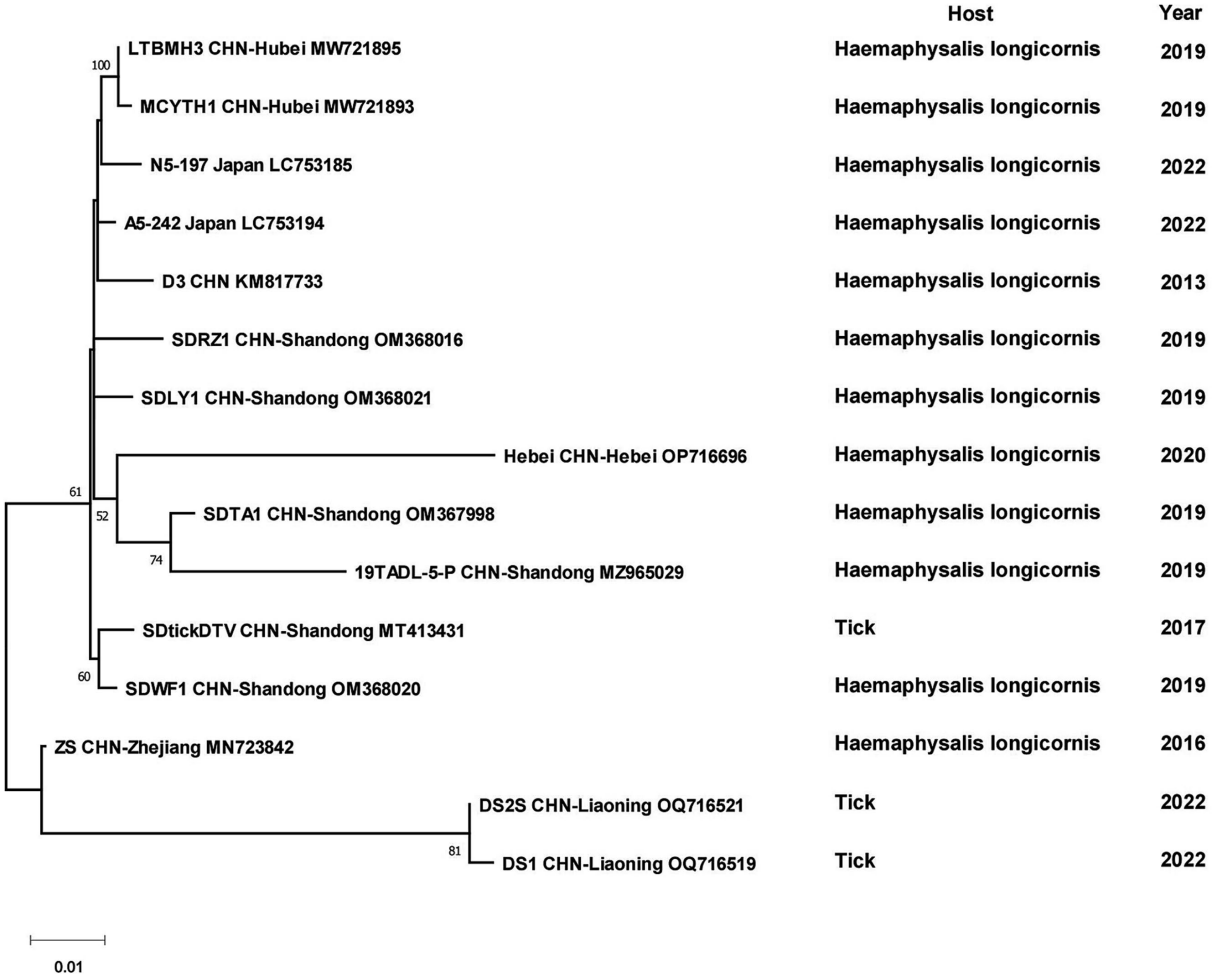
Phylogenetic tree of the S segment of Dabieshan tick virus.

The sequences of 131 SFTSV strains from China and 6 strains from other countries formed two separate branches, one branch was full of Chinese strains, while the other one had some Chinese strains branching with Japanese and South Korean strains. The first cluster could be viewed as five separate branches, I included isolates from Shandong, Henan and Hubei Provinces, II included strains from Zhejiang Province, III included several strains from Zhejiang, Jilin and Liaoning Provinces, IV and V were clusters of strains from Jiangsu and Henan Provinces, respectively. The other branch included strains from Shandong, Henan and Zhejiang Provinces with strains from Japan, Korea and Thailand, in which four strains from Zhejiang Province were clustered with foreign strains. Although sampling times or hosts were different, strains geographically from the same province always clustered together. Some strains from Henan and Shandong Provinces were scattered in the phylogenetic tree (Figure 5). Among the DTV strains, those found from Shandong and Hubei Provinces and Japan formed a larger cluster. DTV found in Liaoning Province in 2022 and Zhejiang Province in 2016 form a distinct branch and did not cluster with the sequences of the strain that was first reported in 2013, especially the Liaoning strains, which were genetically far from the rest strains (Figure 6).

**Figure 7:**
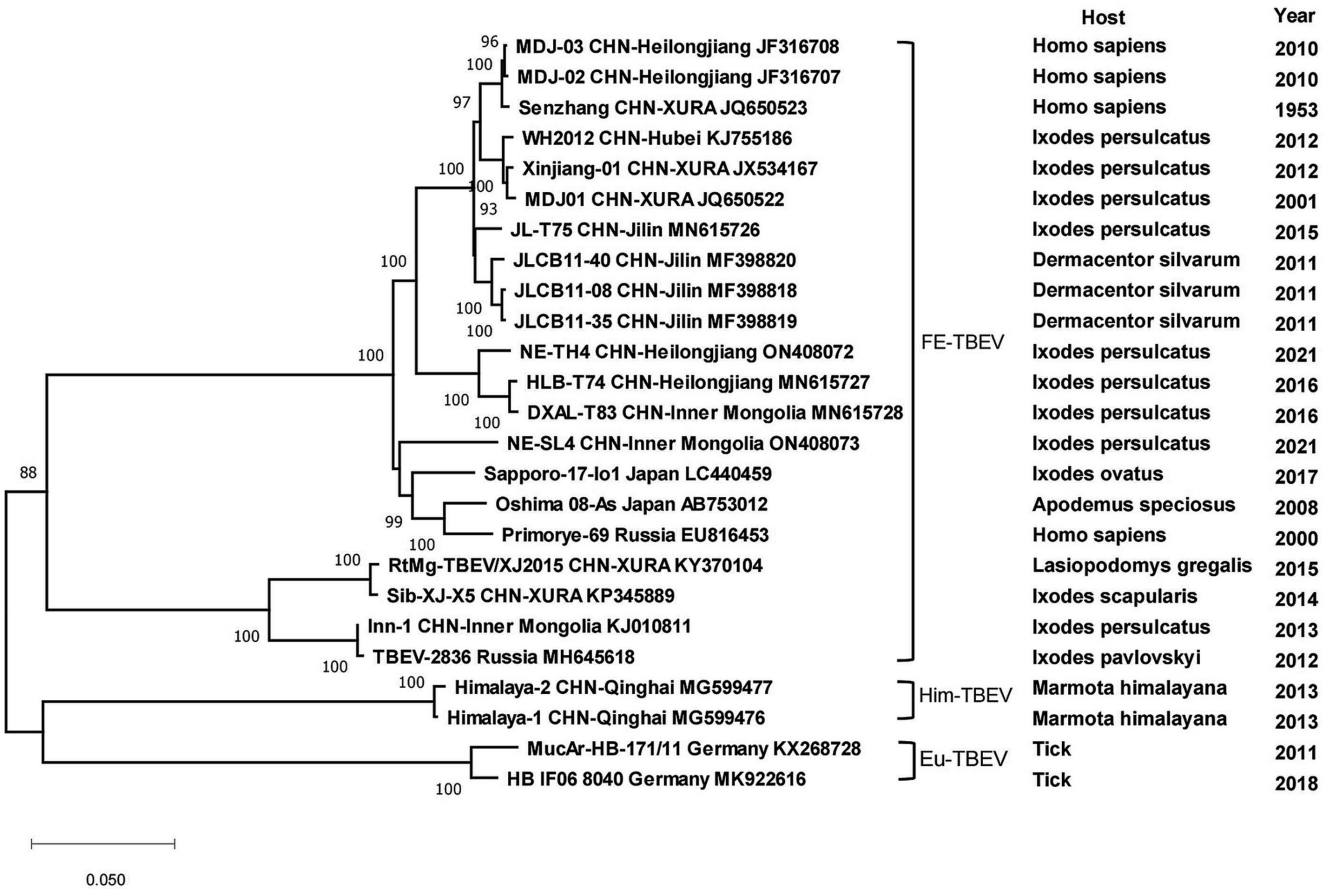
Phylogenetic tree of the complete genome of Tick-borne encephalitis virus.

**Figure 8:**
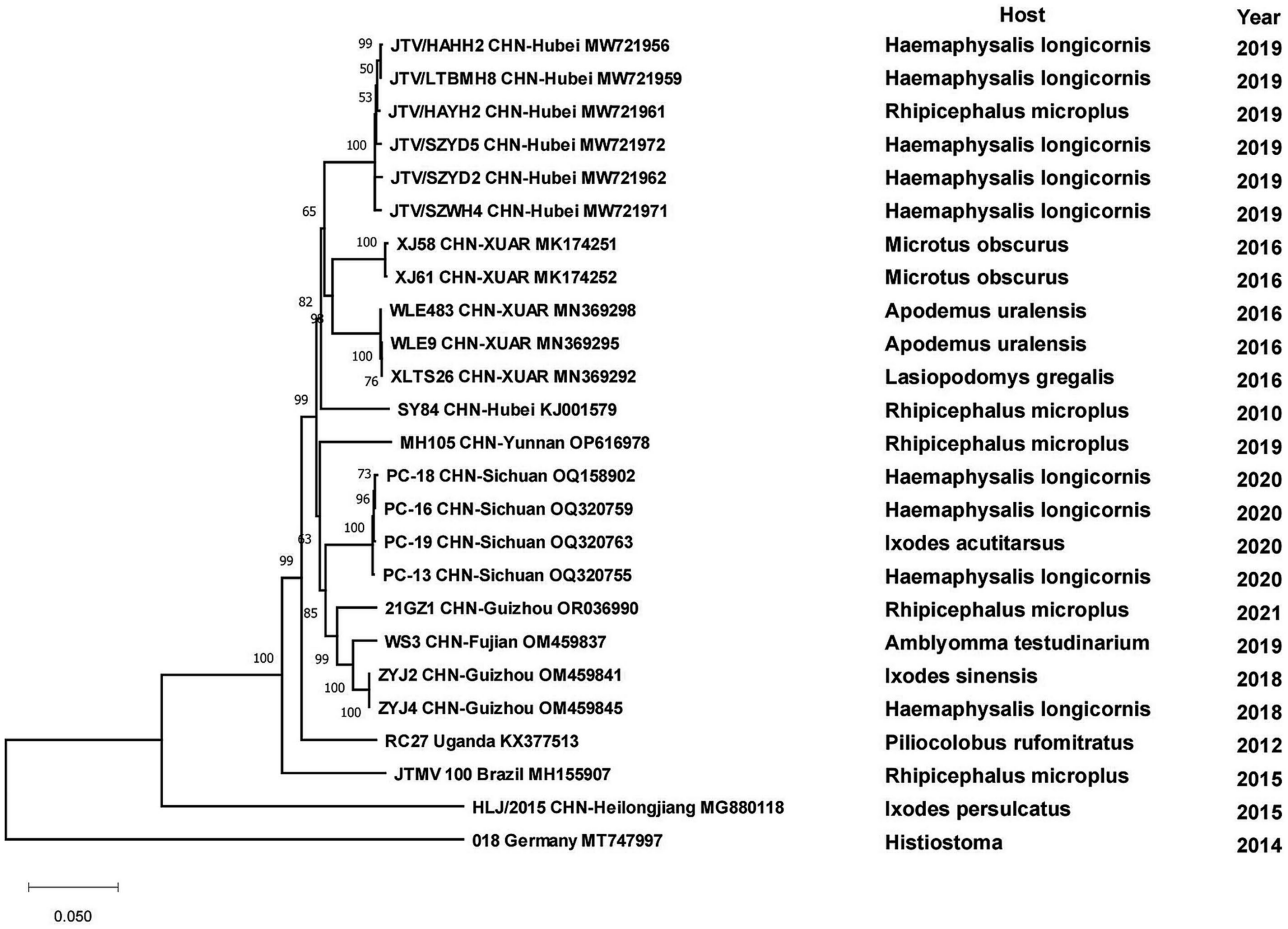
Phylogenetic tree of the segment 1 of Jingmen tick virus.

All strains of tick-borne encephalitis viruses in China belonged to the TBEV-FE, except for two Himalayan TBEV strains detected in Qinghai Province in 2013. Three strains collected from *I. persulcatus* in Inner Mongolia Province with sampling dates of 2013, 2016 and 2021 formed clusters with strain TBEV-2836 isolated from *I. pavlovskyi* in Russia in 2012, strain HLB-T74 from Heilongjiang Province and strains from Japan and Russia, respectively, and they were in three clusters of different branches. Strains detected from human samples in 2010 in Heilongjiang Province clustered with strains from human samples in XUAR, whereas strains detected in *I. persulcatus* in 2016 were genetically closely related to strains detected in *I. persulcatus* in Inner Mongolia in the same year, and the two types of sequences from Heilongjiang Province were genetically distantly related. As for strains from XUAR, strains detected in *I. persulcatus* in 2001 and 2012 clustered with WH2012 from Hubei Province, but the strains detected in *I. scapularis* and *Lasiopodomys gregalls* formed a cluster with Inn-1 and TBEV-2836 and were distant from several other strains (Figure 7). The sequences of JMTV in China were divided into two branches, one was the sequences of XUAR and Hubei Province, which were clustered together regardless of their hosts, and the other was the sequences of Yunnan, Guizhou and Sichuan Provinces, and other places in southwesten China. However, the JMTV sequence found in *I. persulcatus* from Heilongjiang Province strayed outside the centralized parts of the phylogenetic tree and did not cluster with other strains in China (Figure 8).

## 4 Discussion

The diversity of TBVs has increased in the twenty-first century with the emergence of new viruses. Heartland virus (HRTV), Bourbon virus, Powassan virus and Colorado tick fever virus are emerging as hotspots for arboviruses in the United States (Rodino et al., 2020). In Europe, in addition to TBEV and CCHFV, several TBVs pose a threat to human health, although few cases have been reported of them, such as Eyach virus, Louping ill virus, Pustyn virus and Sulina virus (Charrel et al., 2004). TBVs were also diverse and widely distributed in China.

According to our statistics, there were at least 8 genera of viruses in 5 families in China, and most of them first appeared in China in the last few years and were detected only in some countries around China. The species of TBVs of the genus *Orthonairovirus* were increasing, such as *Tamdy orthonairovirus*, which had similar clinical symptoms. GTV of the family *Phenuiviridae* and ALSV of the family *Flaviviridae* were both new tick-borne viruses in recent years. Most of these emerging tick-borne viruses were detected based on surveillance for tick-borne diseases in China, and when researchers were able to isolate a virus in patients, examination of ticks collected in the patient’s place of residence, as well as animals such as cows and sheep, were also partially positive for the virus, suggesting that the virus were spreading in the local ecosystem, which would make the local population at risk of exposure to viral infections. In addition to those newly identified viruses, many TBVs existed but have not been reported in China previously, such as NSDV, TAMV, YEZV, MKWV, and KSIV. NSDV was previously thought to be endemic only in East Africa, but some studies have shown that the Ganjam virus is an Asian variant of NSDV and has been found in India and Sri Lanka. This demonstrated that Asia was also becoming endemic for NSDV. It is notable that the earliest strain of NSDV in China was different from strains from other Asian areas and Africa, and was considered to be a new strain (Marczinke and Nichol, 2002; Gong S et al., 2015; Gong Z et al., 2015). Alternatively, some TBVs could be imported exogenously from other countries and regions. Transmission of tick-borne pathogens across regions was not unusual, with migratory birds being one of the main contributing factors (Hasle, 2013; Hornok et al., 2016). In focusing on emerging TBVs, it is important to identify them from closely related viruses of the same viral family. Bai et al. found that the epidemic of KSIV can lead to misinterpretation of the high seroprevalence of TBEVs, which can cause misjudgment of the prevalence of TBVs and the degree of risk to humans (Bai et al., 2022). In addition to the proliferation of virus species, the vectors of some TBVs also changed such as CCHFV, TBEV and SFTSV, especially the species of ticks carrying SFTSV increased significantly. TBEV was the predominant TBV in Europe and was distributed in at least twenty-five European countries. In contrast to China, *Ixodes ricinus* was the most important vector for the transmission of TBEV in Europe. While *I. persulcatus* mostly played its role in transmitting TBEV in the forests of Siberia and Russia. The increasing number of tick-borne virus species and hosts in China has led to a rapid epidemic of tick-borne viral diseases in humans.

In this study, the distribution of TBVs was predominantly in the North of China, especially in the northeastern region and XUAR, which is similar to the general distribution of tick-borne diseases in China by Wu et al. (2013). The geographic distribution of TBVs for each virus family and the distribution of vectors of each virus were mapped. Something was interesting when combining them. The number of *Hy. asiaticum* was the most dominant factor influencing the distribution of CCHFVs in the previous study (Teng et al., 2022). Moreover, among the environmental factors predicting the distribution of *Hy. asiaticum*, desert grassland cover and rainfed farmland cover were the main factors (Zhao et al., 2021). Predictions in some articles indicated that Gansu, Ningxia, Shaanxi, and Yunnan Provinces were risk areas for the occurrence of CCHFV, and Yunnan Province had found CCHFV in ticks, whereas Gansu and western Inner Mongolia Provinces were neighbors to the XUAR, with similar landscapes and climates, more desert or bare land, and people engaged in farming, but there had no reports of tick-borne CCHFV or human infections with CCHFV. The reasons need to be studied in the future or the surveillance of tick-borne CCHFV and unidentified febrile illnesses should be strengthened in these regions. Although TCTV-1 and TCTV-2 were in different virus families, they had the common characteristics of higher positivity in ticks of the *Dermacentor* spp. than the *Hyalomma* spp. (Zhao et al., 2021). Among the environmental factors affecting distribution, both grass cover and seasonal precipitation had a greater impact on the *Dermacentor* spp., which might explain why most of TCTV-1 and TCTV-2 were detected in XUAR around the Tianshan Mountains. SGLV was mainly transmitted by *I. persulcatus* and *I. crenulatus* distributed in Northeast China and XUAR. The potential for *I. persulcatus* and *I. crenulatus* in XUAR to carry SGLV and transmit it to humans was noteworthy because SGLV had been found in local rodents. In a prediction model of SFTSV, the number of *H. longicornis* and altitude had a greater effect, with lower altitude resulting in more SFTSV cases, while the distribution of *H. longicornis* was related to precipitation and mean annual temperature. This might be related to the fact that SFTSV cases were mainly in central China, which had a lower elevation and higher precipitation than the northwestern and southwestern China (Miao et al., 2020). *H. longicornis* was distributed throughout Shaanxi Province where the precipitation and climate were also similar to central China, the reason for fewer reports of SFTSV in Shaanxi Province might be the high altitude. The occurrence of TBEV was closely related to meteorological factors, such as the presence of *I. persulcatus* and mixed broadleaf-conifer forest (Sun RX et al., 2017). In agreement with previous studies, the majority of TBEV in China was distributed in the forested areas of the Great Khingan Mountain, the Lesser Khingan Mountains, the Changbai Mountains and the Tianshan Mountains in XUAR, which was consistent with the distribution of *I. persulcatus*. However, when preventing SFTSV and TBEV infections in populations, the specificity that they can be transmitted directly from person to person without insect vectors should be emphasized. TBEV can be transmitted to humans by drinking raw milk from infected animals, and it has also been found that TBEV may be transmitted from infected mothers who have not been immunized with the TBE vaccine to their offspring through breast milk (Kerlik et al., 2022). It suggested that preventive measures cannot be taken against its vectors alone. The majority of JMTV was distributed in the plains of central China, which might be related to its higher detection in *H. longicornis*. There were no studies on the prediction of JMTV distribution at present. Most of the TBVs had some characteristics in their distribution areas or vector tick species, but JMTV had no specificity probably because of the large number of tick species that could transmit it, so it could not be analyzed what factors had a greater impact on its distribution. PIV5 and LCMV, which appeared in China for the first time, were carried by the *I. persulcatus, D. nuttalli* and *D. silvarum*. Both of these two viruses could be found in the forested areas of the Northeast and the Tianshan Mountains, which was also a cross-distribution area for their vectors. These areas might be potential regions of infection for the PIV5 and LCMV (Vilibic-Cavlek et al., 2021). LCMV was a natural teratogen that could be transmitted to the fetus via vertical transmission. It was more dangerous for a pregnant woman if infecting LCMV, which could result in fetal miscarriage, intrauterine death, or severe central nervous system disease. The risk of human infection of LCMV should be assessed by monitoring the prevalence of LCMV in natural rodents and ticks in northeastern China. Animal reservoirs should not be ignored when exploring the factors influencing the distribution of TBVs. The abundance of animal reservoirs had a strong effect on the abundance of ticks. Moreover, the migration of animal reservoirs, either active or passive, led to the geographical expansion of tick populations, which has important implications for the transmission of TBVs between different areas.

All CCHFV strains included in this paper also clustered into some geographically related branches, which was constant with previous phylogenetic analyses (Zhou et al., 2013a). Two of the strains in XUAR that were both detected in 2017 formed a separate Asia branch with the Pakistan strain. It could be concluded that these two strains in XUAR might be imported from Pakistan or spread from XUAR to Pakistan and that they might share a common ancestor with the clustered European strains. At least two different types of CCHFV genotypes currently exist in China. A new strain of NSDV, which was different from other strains in Asia, has only been detected in ticks and sheep in China, but its potential hazards to humans cannot be ignored because of the unknown pathogenicity. A previous study divided the SFTSV strains in China into five genotypes, whereas in this study all strains were divided into two different clusters, suggesting that there might be two types of SFTSV genotypes with large differences in China currently (Yoshikawa et al., 2015). Several strains from Shandong and Zhejiang Provinces were clustered with Japanese and Korean ones, as was shown in other studies. The reason might be that Shandong Province and Japan were located closely together. HRTV from the genus *Bandavirus*, which was detected only in the United States, was clinically similar and phylogenetically related to SFTSV. It was not considered in the phylogenetic analysis of SFTSV in this paper, but the association between them cannot be ignored in the differential diagnosis of SFTSV. The two branches of the phylogenetic tree indicated that the DTV in China could be divided into two genotypes. The phylogenetic tree of JMTV in China was geographically clustered except for the strain from Heilongjiang Province. As fewer studies were conducted on JMTV in northeast China, there were no other strains for reference to identify whether there are new genotypes or not. One strain of TBEV from Inner Mongolia Province was genetically related to the Russian strain and clustered with two strains from XUAR, both of which had different source hosts, and it could be hypothesized that it was transmitted from Russia to Inner Mongolia Province and then spread to XUAR since they border each other and the Russian strains were sampled the earliest.

The limitation of this study is that only published records of TBVs in the literature are available, some areas where TBVs were endemic but not reported may be missed. In addition, the data analyzed in this article was from different literature and the homogeneity of the samples was not ensured when compared them together.

## 5 Conclusion

Eighteen TBVs that could cause serious diseases or potential harm to humans or animals existed in China. XUAR and Northeastern China had more TBVs which should be paid more attention to. There were at least five genera of ticks that could carry or transmit TBVs in China. In addition, several strains of DTV and JMTV that appeared in recent years were new genotypes. The complex situation of TBVs in China brought great challenges to public health. It is vital to strengthen TBV surveillance and build a comprehensive surveillance network in China.

## Data availability statement

The original contributions presented in the study are included in the article/Supplementary material, further inquiries can be directed to the corresponding author.

## Author contributions

YW: Formal analysis, Visualization, Writing – original draft. QZ: Data curation, Writing – original draft. MM: Investigation, Writing – original draft. HC: Data curation, Writing – original draft. RQ: Conceptualization, Supervision, Writing – review & editing.

## Footnotes

^1^http://www.geodata.cn
