# Supplementary material for: Diversity of species and geographic distribution of tick-borne viruses in China
Source: Front Microbiol. 2024 Feb 27;15:1309698. doi: 10.3389/fmicb.2024.1309698 (PMC10929907; doi:10.3389/fmicb.2024.1309698)
Supplement: Supplementary file 1 [file Data_Sheet_1.docx]

**Supplementary Table 1** Tick-borne virus identified by high-throughput sequencing in China

| Family | Genus | Species | Vector | Geographical distribution in China |
| --- | --- | --- | --- | --- |
| *Nairoviridae* | *Orthonairovirus* | Huangpi Tick Virus 1 | *H. longicornis* | Hubei, Liaoning, Gansu |
|  |  | Pustyn virus | *I. persulcatus* | Heilongjiang |
|  |  | Meihua Mountain virus | *unclassified Ixodida* | Fujian |
|  |  | Sichuan tick nairovirus | *unclassified Ixodida* | Sichuan |
|  |  | Gakugsa tick virus | *I. persulcatus* | Heilongjiang, Jilin |
|  | *Uukuvirus* | Tacheng uukuvirus | *Hy. marginatum, D. marginatus, Hy. asiaticum, D. silvarum, D. nuttalli* | Inner Mongolia |
|  |  | Ji'an nairovirus | *H. japonica, H. concinna* | Jilin |
|  |  | Yichun nairovirus | *I. persulcatus* | Heilongjiang |
|  |  | Dali_Nairo_tick_virus | *H. montgomeryi* | Yunnan |
|  |  | Yanbian_Nairo_tick_virus | *H. concinna* | Jilin |
| *Circoviridae* | *Circovirus* | Tick associated circovirus 2 | *H. concinna* | Heilongjiang |
|  |  | Tick circovirus | *H. longicornis, I. crenulatus* | Heilongjiang, Sichuan |
| *Sedoreoviridae* | *Orbivirus* | Guangdong tick orbivirus | *R. microplus* | Guangdong |
| *Spinareoviridae* | *Coltivirus* | Colorado tick fever virus | *I. persulcatus* | Heilongjiang |
|  | *unclassified Spinareovirinae* | Lishui pangolin virus | *A. testudinarium* | Guangxi, Zhejiang |
| *Orthomyxoviridae* | *Quaranjavirus* | Guangdong tick quaranjavirus | *R. sanguineus* | Guangdong |
|  |  | Zambezi tick virus 1 | *R. sanguineus* | Fujian |
| *Phenuiviridae* | *Phlebovirus* | Brown dog tick phlebovirus 1 | *R. sanguineus* | XUAR, Zhejiang |
|  |  | Bole Tick Virus 1 | *Hy. asiaticum, R. turanicus, Hy. scupense* | XUAR, Gansu, Inner Mongolia |
| *Flaviviridae* | *Uukuvirus* | Guangxi tick virus | *A. javanense* | Guangxi |
| *Hepeviridae* | *Uukuvirus* | Sichuan tick hepe-like virus | *unclassified Ixodida* | Sichuan |
| *Nodaviridae* | *Uukuvirus* | Tianjin Nodav tick virus | *H. longicornis* | Tianjin |
|  |  | Yanbian Nodav tick virus | *D. silvarum* | Jilin |
|  |  | Luoyang Nodav tick virus | *H. longicornis* | Henan |
| *Picornaviridae* | *Uukuvirus* | Beijing Picor tick virus 1 | *H. longicornis* | Beijing |

**
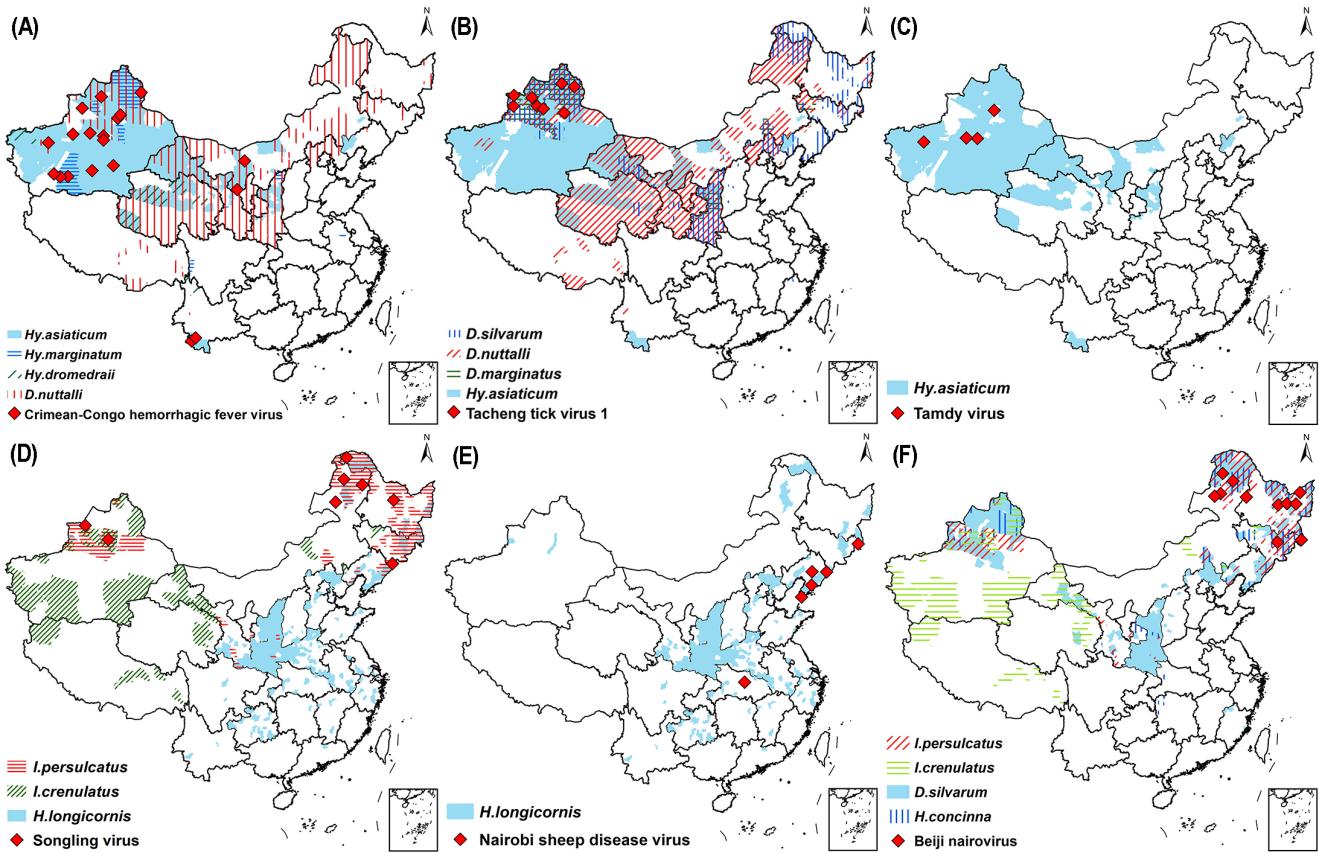
**

**Supplementary Figure 1.**Vector distribution of tick-borne viruses of the family *Nairoviridae*. (A) Crimean-Congo hemorrhagic fever virus, (B) Tacheng tick virus 1, (C) Tamdy virus, (D) Songling virus, (E) Nairobi sheep disease virus, (F) Beiji nairovirus.

**
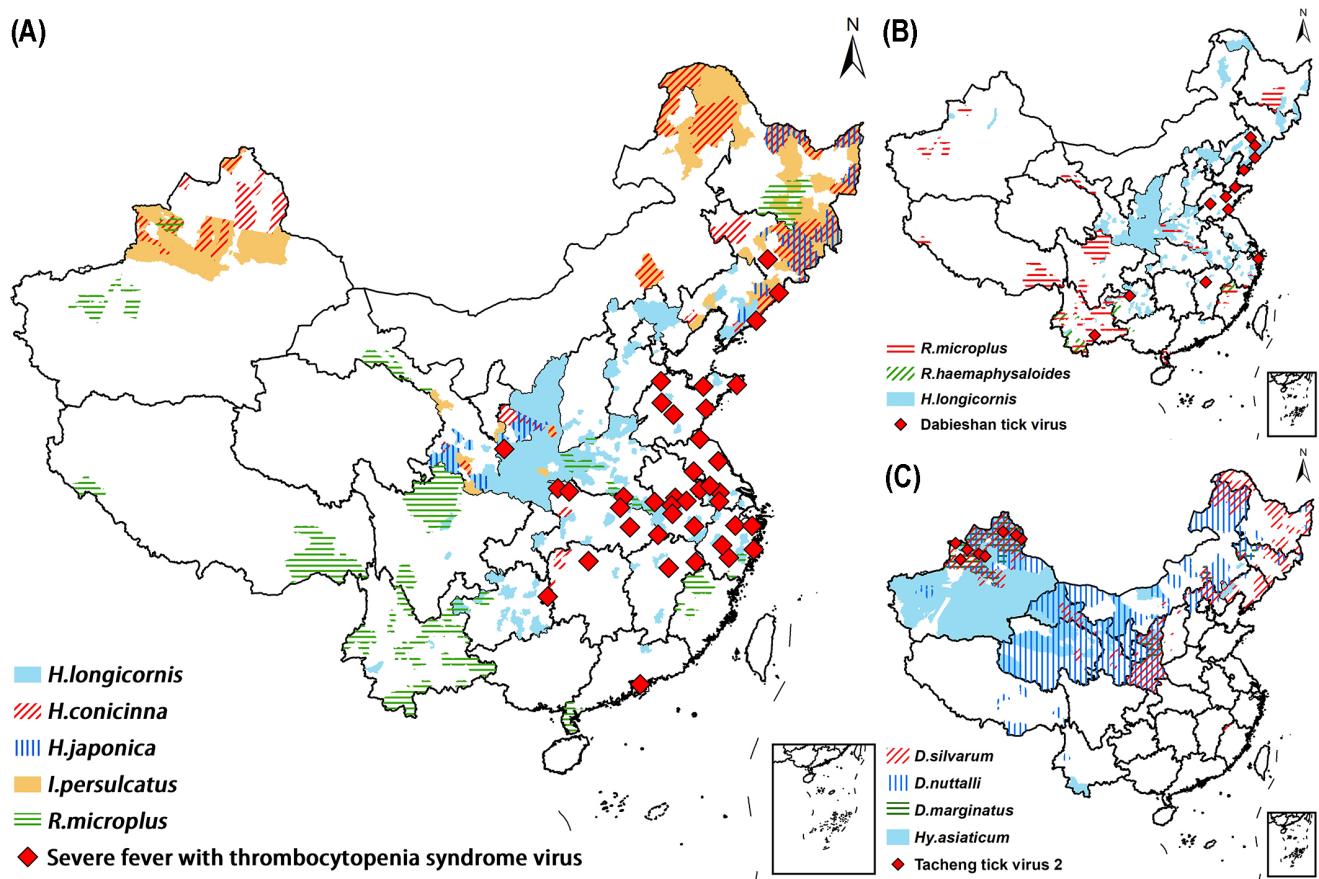
**

**Supplementary Figure 2.** Vector distribution of tick-borne viruses of the family *Phenuiviridae*. (A) Severe fever with thrombocytopenia syndrome virus, (B) Dabieshan tick virus, (C) Tacheng tick virus 2


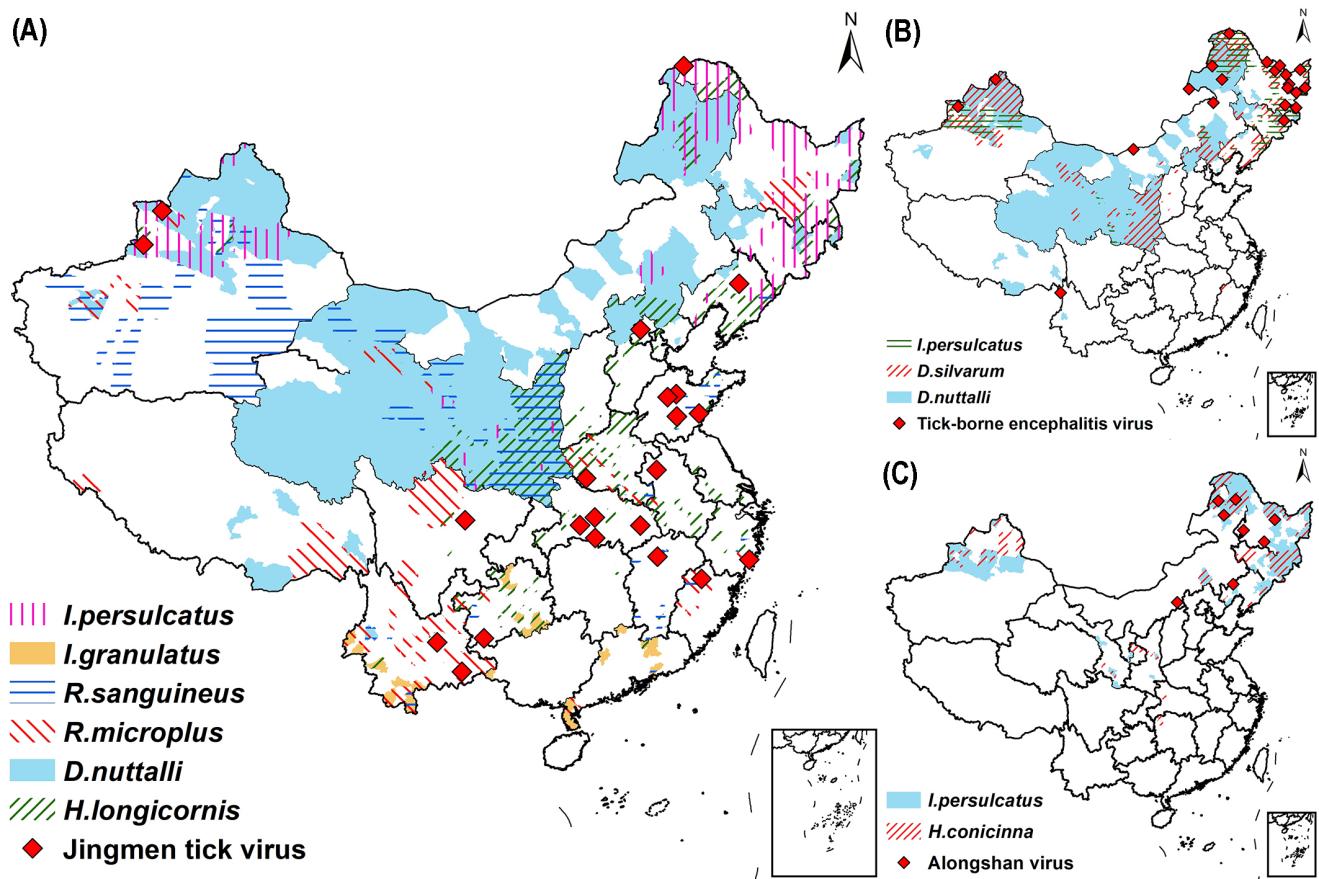


**Supplementary Figure 3.** Vector distribution of tick-borne viruses of the family *Flaviviridae*. (A) Jingmen tick virus, (B) Tick-borne encephalitis virus, (C) Alongshan virus

**
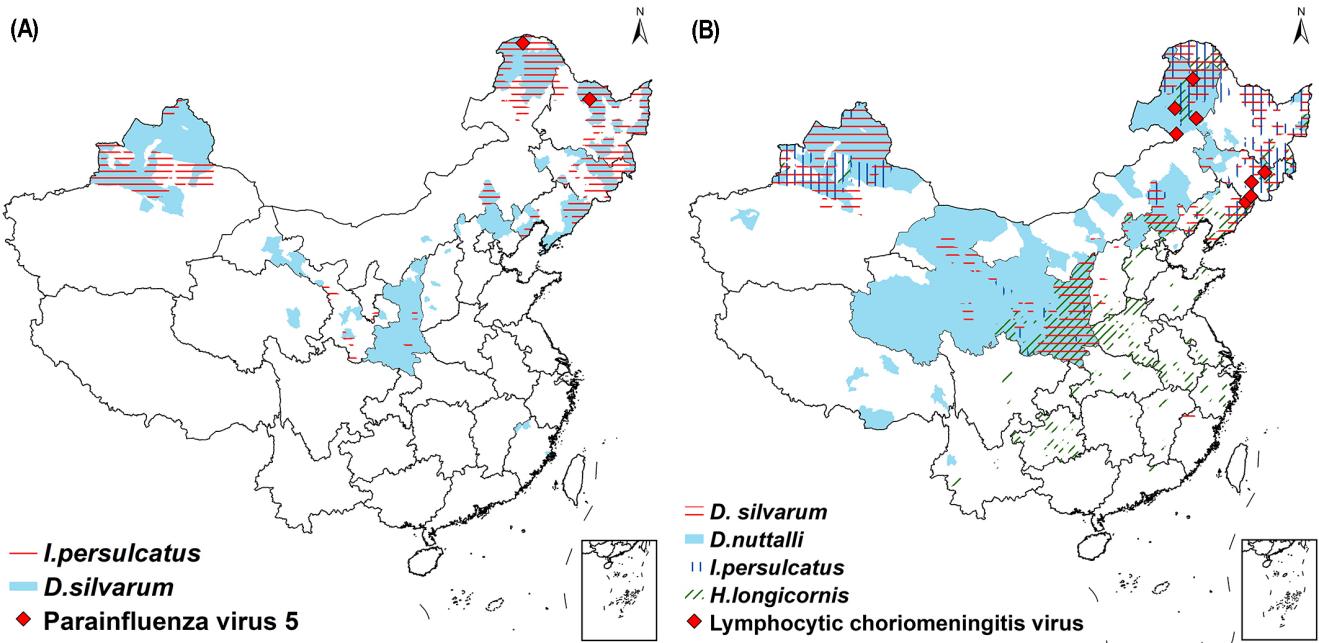
**

**Supplementary Figure 4.** Vector distribution of tick-borne viruses of the family *Paramyxoviridae* and *Arenaviridae*. (A) Parainfluenza virus 5, (B) Lymphocytic choriomeningitis virus
